# The Michigan men’s diabetes project: perspectives on a peer-led diabetes self-management and support intervention for Black men with type 2 diabetes

**DOI:** 10.1186/s12913-024-11884-2

**Published:** 2024-12-18

**Authors:** Alana M. Ewen, Jaclynn M. Hawkins, Katherine A. Kloss, Srijani Sengupta, Hannah Burgess, Robin Nwankwo, Martha Funnell, Jamie Mitchell, Gretchen Piatt

**Affiliations:** 1 Department of Behavioral and Community Health, School of Public Health, University of Maryland, 4200 Valley Dr., Suite 1234, College Park, MD 20742 USA; 2 School of Social Work, University of Michigan, Ann Arbor, MI USA; 3 Department of Learning Health Sciences, School of Medicine, University of Michigan, Ann Arbor, MI USA

**Keywords:** Black men, Type 2 diabetes, Self-management education/Support

## Abstract

**Background:**

Black men are more likely to be diagnosed with type 2 diabetes (T2D) compared to non-Hispanic White men, especially those over 55 years of age. Although there is ample evidence around the efficacy of peer-led diabetes self-management and support (PLDSMS) programs in improving diabetes health outcomes, Black men living with T2D experience several barriers to meaningful participation in peer-led programs and program developers face barriers to implementation. This qualitative study aimed to identify perspectives from collaborators on barriers and facilitators that impact the implementation of a PLDSMS intervention for older Black men with T2D.

**Methods:**

Qualitative data were collected as part of the Michigan Men’s Diabetes Project. We used the Tailored Implementation in Chronic Diseases (TICD) Checklist to construct the semi-structured interview guide. TICD domains served as themes. Codes were later generated as a team (*N* = 3) from chunks of related text. Eight 1-on-1 semi-structured interviews (two researchers, three peer leaders, one community collaborator, two certified diabetes care and education specialists) were conducted between April 13–22, 2022 via Zoom. We engaged in thematic content analysis and used the rigorous and accelerated data reduction (RADaR) technique and Rapid analysis.

**Results:**

Themes included guideline factors; individual collaborator factors; patient factors; professional interactions; incentives and resources; capacity for organizational change; and use of technology. Guidelines for implementing a PLDSMS program for Black men with T2D are lacking. For effective implementation, collaborators need interpersonal and session facilitation skills, flexibility, and cultural awareness. Although Black men with T2D may initially be apprehensive about participating in a PLDSMS program due to lack of knowledge, masculine norms, and stigma, these programs offer a safe space, a sense of brotherhood, and transparency. Having a physician champion is key in supporting organizational changes needed to implement PLDSMS programs in health systems, particularly as PLDSMS is not currently a billable service.

**Conclusions:**

The PLDSMS program is culturally relevant in engaging older Black men with T2D. In addition to building trust among participants, successful development and implementation of a peer support program requires flexibility and tailored communication strategies. Findings can be used to inform future iterations of PLDSMS programs.

**Supplementary Information:**

The online version contains supplementary material available at 10.1186/s12913-024-11884-2.

## Contributions to the literature

- Perspectives from a diverse range of individuals create a more holistic understanding of implementation challenges and catalysts and may help guide healthcare providers and organizations in the quest for successful PLDSMS program delivery.
- Findings suggest that comprehensive guidelines and tailored strategies are needed to effectively implement PLDSMS programs in health systems, particularly for high-risk, vulnerable populations that face an undue health burden.
- This study adds value to the implementation science literature by expanding our understanding of how best to integrate peer-led models in community-based clinics, all while acknowledging the importance of physician champions.

## Introduction

The prevalence of type 2 diabetes (T2D) in the United States (US) remains high, and its consequences are life altering for impacted individuals and their families [1]. Over 36 million individuals in the US are living with T2D, representing 11% of the population [2]. Racial disparities remain evident. Black Americans have a higher prevalence of diabetes than non-Hispanic White Americans, 12.1% and 6.9%, respectively, and men have a higher prevalence than women, 12.6% and 10.2%, respectively [3]. Black Americans are also more likely to experience diabetes-related complications. Among US adults with diagnosed diabetes, 23.1% of non-Hispanic Black adults had moderate to severe chronic kidney disease, while 17.2% of non-Hispanic White adults experienced this complication [3]. Although T2D has increased in younger groups [4], older Black men (over 55 years of age) constitute a large percentage of individuals affected by diabetes-related complications due to self-management challenges and other barriers [5]. These challenges may be addressed via participation in peer-led diabetes self-management and support (PLDSMS) programs. While limited, literature suggests that Black men prefer peer support to help address health concerns, and are enthusiastic about participating if accessible [6, 7]. Peer-led support interventions may be represented in various forms, including group and individual informal support. Generally, connecting with peers who have similar experiences creates a safe space for discussion and camaraderie, which can be therapeutic [8]. Program content that is relatable and that provides anonymity within the private group setting also contributes to participants’ comfort in sharing their thoughts and opinions with peers [8].

PLDSMS programs that meet the needs of the target population and are aligned with the community’s resources, show great feasibility and success in reaching adults living with diabetes [9]. Peer support programs effectively lead to positive changes in participant behavior and improved diabetes management outcomes such as increased glycemic stability, blood glucose self-monitoring, and reduced diabetes distress [9, 10]. However, despite the significant benefits from participation in peer support programs, recruiting and retaining Black men is challenging, with Black men making up less than 10% of participants [6], and older Black men participating at lower rates than women and other racial and ethnic groups [5].

One known barrier Black men living with T2D face to meaningful participation in diabetes self-management programs is the stigma associated with seeking help, which is tied to masculine norms, and which peer-led programs inherently address [11, 12]. Stigma largely discourages men from seeking help for mental and/or physical needs [13–15]. In a systematic review, peer-led interventions were found to effectively reduce self-stigma and related stress by positively impacting personal recovery (self-efficacy and seeking help) [16]. Furthermore, innovative online behavioral health interventions, such as the Young Black Men, Masculinities, and Mental Health (YBMen) Project, a tailored psychoeducational intervention, have successfully leveraged virtual methods, specifically social media, to support Black men’s well-being, promoting positive masculinity and increased social support [8].

Many of these studies have shown great promise in disease management, but participants are often younger (under 50 years of age) and/or the racial composition is often diverse, emphasizing the importance and gap in our understanding of the efficacy of peer support interventions for older Black men [8, 16]. Health promotion interventions targeting Black men over 55 years of age remain limited, and little is known about the facilitators and barriers of PLDSMS program uptake for this population.

The purpose of this qualitative study is to understand the structural, contextual and cultural factors impacting the implementation of a PLDSMS intervention adapted for Black men with T2D in the US. Briefly, the PLDSMS program targeted Black men 55 years of age and older living in the Metro Detroit area with a clinical diagnosis of T2D. Through collaboration with peer leaders, the program aimed to improve participants’ hemoglobin A1c levels and self-management behaviors. Virtual sessions were designed to provide diabetes education and ongoing support. Additional detail can be found elsewhere [17]. Understanding determinants of implementation and adoption of PLDSMS programs tailored to Black older men who are at higher risk of T2D and its complications, is critical for improving diabetes self-management programs and health outcomes among Black men with T2D. We aim to use the qualitative data to improve future programs for Black men with diabetes.

## Methods

### Study design

The study was conducted as part of the Michigan Men’s Diabetes Project using the Tailored Implementation in Chronic Diseases (TICD) Checklist [18]. The TICD is a comprehensive framework that describes the determinants of implementation success [18]. Determinants of practice are the barriers and facilitators that may impact the implementation of an intervention. Utilization of this framework allows for the adaptation of future PLDSMS interventions that account for identified challenges and enablers of change, ensuring appropriate tailoring for older Black men living with T2D.

### Participants and recruitment

Two Certified Diabetes Care and Education Specialists (CDCESs), one community collaborator (CC), three peer leaders (PLs) from previous studies, and two researchers in the fields of diabetes and/or men’s health and physical health were recruited via email using convenience sampling. All participants were part of prior studies and/or had been trained by prior staff, and thus had been aware of the interviewer’s personal interest in the research topic, as well as the goals and objectives of the study.

### Interview guide development

Deductive reasoning was used in the development of the interview guide (Additional File 1), where domains, derived from the TICD Checklist, served as themes. The TICD Checklist includes 57 potential determinants of practice grouped into seven domains: guideline factors; individual collaborator factors; patient factors; professional interactions; incentives and resources; capacity for organizational change; and use of technology. For this study, patient factors referred to factors unique to Black men with T2D. “Use of Technology” was added as a domain, replacing “Social, political, and legal factors,” which was not relevant to this study. Technology could aid in the delivery of peer-support programs, particularly since it has become more widely adopted in the face of public health challenges. We aimed to investigate how technology could best be integrated into PLDSMS programs. The questions for this domain were formulated by members of the research team who had expertise in this area.

### Data collection

Eight one-time, 1-on-1 semi-structured interviews were conducted via Zoom between April 13–22, 2022 by a member of the research team (JMH). The interviewer, who is also the Principal Investigator (PI), self-identifies as an African American female and is an Associate Professor with PhD training in social work and sociology. She has several years of experience in diabetes health disparities research and in developing and adapting interventions for Black men with T2D. Interviews were conducted in a private, quiet location of the participants’ choosing to better understand factors that may impact the implementation of a PLDSMS intervention adapted for Black men with T2D in the US. Interviews were approximately one hour in duration. Following completion of the interviews, JMH created field notes, and participants received a USD 30 gift card.

### Data analysis

Thematic content analysis, an iterative process, was used to analyze the data. Part of this process entailed understanding how the data fit into pre-identified themes. Given its flexibility, refining themes may be necessary if misalignment is detected. The analytic team generated codes from chunks of related text using inductive reasoning. The team initially consisted of three data coders (AME, KK, SS). Two coders (AME, SS) later met with HB to conclude the coding process after KK transitioned to a new position. SS transcribed all eight interviews. The team used a combination of the rigorous and accelerated data reduction (RADaR) technique and Rapid analysis, which allowed for a quick understanding of major qualitative findings from the interviews [19, 20]. This process also facilitated the development of a coding scheme informed by the depth and breadth of data in each domain [19, 20]. Rapid analysis has been applied in prior research using the TICD framework [21]. Journaling and memoing were performed independently by members of the research team.

All analyses were conducted in Excel using Data Analysis Reduction Tables (DARTs), a structured method to systematically organize and analyze data. Each member of the research team independently generated their own codes. This process was subsequently followed by a meeting with the team to discuss the codes. Text chunks from each of the interviews were re-read to further refine the codes. Irrelevant text from the interviews was omitted. As a team, focused coding (1–3 words) was then initiated for the PLs and CC, followed by open and focused coding for all participants. Through this method of analysis, the team became more immersed in the data.

After analyses of all transcripts, an adjudication meeting was held to discuss the codes each member generated. AME re-read all interviews, starting with the PLs. The team then met to share perspectives and interpretations. The team began with the CC and PL1 interviews, reading through the text chunk lines and subsequently generating themes. The long-term goal was to generate codes based on roles and then collate these codes across all roles. TICD domains and determinants were cross-referenced to identify whether there was any alignment.

Each team member came in with a different perspective. KK personally knew many of the interviewees through her community work and mentioned needing to check her bias at times throughout the coding sessions. SS was also familiar with the interviewees, whereas AME had no prior interaction with any of the participants, which reduced her bias during the analysis phase.

Study data were stored in Google Drive at the University of Michigan, a secure cloud-based storage service that encrypts files upon upload [22]. The COnsolidated criteria for REporting Qualitative research (COREQ) guidelines were followed. Individuals were asked to provide informed verbal consent prior to participating. The study protocol and informed consent procedure were approved by the University of Michigan Medical School Institutional Review Board (IRBMED) HUM00200496.

### Reflexive position statement

At the time of analysis, the lead author was a second-year PhD student at an external institution and did not have first-hand knowledge of the environmental exposures Black men with T2D face while living in the Metro Detroit area. However, as a Pre-Doctoral Fellow at the institution where the study was conducted, she had the opportunity to learn more about the PLDSMS program for Black men. In addition, a member of the research team has a family member who is living with T2D, identifies as a Black male and has had adverse experiences due to living with the chronic condition. The team member’s background allowed them to assess the interview questions and corresponding responses more critically and effectively, but given their closeness with the health condition and the demographic group, their interpretation of the data may have been biased.

## Results

All eight invited participants elected to participate in the study. Participants ranged in age from 45 to 70 years. Most (75%) self-identified as African American/Black and most (63%) were male. Seven themes were identified prior to analysis and included: (1) Guideline Factors; (2) Individual Collaborator Factors; (3) Patient Factors; (4) Professional Interactions; (5) Incentives and Resources; (6) Capacity for Organizational Change; and (7) Use of Technology (Table 1).

**Table 1:** Tailored Implementation in Chronic Diseases (TICD) Domains and Sample Interview Questions

Domains^a^	Sample Questions	Text Chunks^b^	Barriers/Challenges (Codes)	Facilitators/Successes (Codes)	Infrastructure/Skills (Codes)
Guideline Factors	Are there guidelines for implementing a PLDSMS program in a medical or community setting?Is PLDSMS appropriate for the culture of medical settings and community settings? Why or Why not?	"...to my knowledge, there are no guidelines... it's a hole in the research that we do...I tend to think of, when we implement a peer intervention or program or whatever I think about those four key functions of peer leaders. You know, to provide support and empathy ... from a broader perspective, I would say that using concepts related to CBPR...make sense." - **Researcher 1** "Community. I feel like where the atmosphere is relaxed...you just in a free place. In a medical center [it’s like] okay [is] this about medical, is this about this, I'm looking at this, and so your mindset goes to what's around you, but [if] you have an open, free environment then you can be free...it opens the door for them to do that because, like I said, it's the women- men it’s just, we just got our own way of stuff... we kind of secretive, ...and I’m saying men period, you know, not like Black men, just men period...culture has taught us that, we can't have emotions, we can't have this, and we can't have that, you gotta be strong, whatever strong supposed to be, you know, [whatever] their definition of strong is. We have all this bottled in us, but when we come to our own it's like, the place that the man is most free at is at a barbershop...as a peer leader you have to make them feel comfortable [and] confident in this class...I'm no better than you, you're no better than me, just because I’m the leader, I'm not over you. This about you, really...We really had a good time..., by the time the end of the class came, people were a little [like], 'ah man, the class is going to end?' You know?...Because we became like brothers." - **PL2**	Lack of guidelines, trust in lay health workers, rigidity, financial restrictions, roles (diabetes educators and peer leaders) need defining, initial apprehensionPLDSMS program setting - if in medical: Needs buy-in, staff availability, lacks emotional support. If in community: Flexibility needed, lacks infrastructure, unstable	Peer leaders provide support, shared identity, empowerment-based, trustPLDSMS program setting - if in medical: infrastructure. If in community: receptive, safe space, relaxed, brotherhood	**Infrastructure:** Availability of space
Individual Collaborator Factors	What skills do [diabetes educators/peer leaders] need to have to implement a PLDSMS program for Black men with type 2 diabetes?	"...they would certainly need to be very interested in people in general..., but they would also need to be somewhat knowledgeable about what diabetes is and the things that it can cause, and have some interest...[They would also have to have] a willingness, of course, to learn." - **PL1** "The first thing I would say...it's listening. And why I say listening, [you have to] listen to the in between the lines. That way you catch where they really at, what their emotion is, what their feeling is...Because you have to reach them where they at... be okay with going off the structure plan...All the work that you guys did to set this up, we don't want to take away from that, but just learn how to blend the two...to get the most out of the men as well as to give you the most out of what you're looking for..." - **PL2** "I believe the type of person that would be successful running a program like this is someone who first and foremost has good communication skills. When I say communication skills, normally people go straight to being able to talk, but I think the most important thing with communication is being able to effectively listen...Being able to be patient because sometimes things will come up, you want to cut somebody off or shut them down or something like that, but just being patient with folks and meeting them where they are...You know, interpersonal skills... As a facilitator, your openness to learning." - **PL3** "I think the skills would be understanding the facilitation process...recognizing that this is a support program, it is not geared for accommodating the accreditation standards...recognizing that there's a distinction between the support and education..." - **CDCES2**	Define roles		**Skills:** Interpersonal, being knowledgeable, willingness to learn, active listening, flexibility, emotional intelligence, facilitation
Patient Factors	Do you think Black men with type 2 diabetes want to receive PLDSMS? Why or why not?Is PLDSMS culturally appropriate for Black men with type 2 diabetes? Why or why not?	Black men wanting to receive PLDSMS: "I think if you ask them they’d say no. You have to have sort of a hook for them. And I think that once I think they would never say yes, but deep in their hearts, they do wonder, because they want to live, I mean, and they want to manage it. So you kind of need a hook, I think, saying it's all for men designed specifically for you, whether you're African American or just a man or random man or whatever, and I think that's that's a bit of a hook. But I also think about what would important for them, why is it important for them to take care of their diabetes, to take care of family to your job?" - **CDCES1** I didn't know the answer to that until your recent study and my understanding is that, yes, the men in in that study wanted a peer led program but they wanted the peer leader to have skills to be able to facilitate effectively meaning asking specific questions being open and transparent. And just knowing, being confident that they knew what they were doing..." - **CDCES2** Culturally appropriate: "Yeah...Strangely enough, it certainly avoids the people shutting up because they don't want to talk about things with people of another race...I think that was a very good thing about doing it with Black men as opposed to all men in general. Most of the guys that I worked with were fairly close, some of them were neighbors or worked at jobs where they worked with other people. The interest level of each group seemed to be very, very closely associated...it’s like you're supposed to be the strong guys there, and you got something here that can tear that down and some of the things that can help that [or] can at least slow down the diabetes itself, which is really the cause of the problems." - **PL1**	Initial apprehension, ignorance, masculine norms, stigma; medical setting - not a safe space, church - dependent on participant comfort	Empowerment-based, culturally tailored messaging, flexible, shared identity, safe space, altruism, skilled peer leaders, interest, brotherhood, transparency	
Professional Interactions	What is the communication process among team members while running a PLDSMS program for Black men with type 2 diabetes?How can peer leaders support Black men with type 2 diabetes to learn about and enroll in PLDSMS?	"...I would say what has worked well… making sure that people have a voice. Everybody,...not just the people doing the study, but the people in the community. I think that we need to be better at understanding what the community needs. What they perceive as important. In comparison to what we think is important. Being transparent around timelines around incentives. If it is a research study, being very, very clear about the research processes...trusting one another, being clear about expectations." - **Researcher 1** "...I think as important, if not much more important, was the monthly sessions among peer coaches..." - **Researcher 2** "...I think that ongoing support from the educator or whoever they're working with whether it's the team, or whoever, you know the project team, they need that ongoing help. Because they're kind of out there on their own. And it is frustrating at times when people keep telling you the same thing, every week and they're still not doing anything or they're asking they're dealing with really impossible issues. And a lot of it is just kind of reminding them that they're not there to fix it... And so it's it's that kind of ongoing, continuing, reminding what you're here for you did the right thing it's that kind of. Its support and confidence." - **CDCES1** "I know we did have a couple of times where we would assess a meeting after we had it, but it wasn’t always, and I think some of it had to deal with the fact that we're on it for an hour and a half, so...and [program manager] was respecting our time, knowing that, you know, we have lives that we're living as well, but maybe you know, a 15 minute debrief after the meetings or prior to, just so that you know we're staying sharp on what we're doing and making sure that we're focused on the goal going into it. That would be the only thing I would kind of recommend, but overall, I think the communication was very effective between the staff and the peer leaders." - **PL3** PL support: "We can talk to men, we can say we went through this program, we could say the things that we have learned, things that we have changed. [We can] talk about [how] there’s help out there for us, but yet at the same time, you could still keep your masculinity, you still keep your privacy, to a sense, you know what I’m saying when I say that? Because it's just amongst us, a smaller group of people versus everybody out there, no one but us that’s in it together, we could talk, we can cover- the support that you have from your other men, I can help you and I can support you, because I’m where you are, and this has helped me...The participants will be your best advertisement because they already been through it and experiencing help and then for people who knows them to see the change that they made because of the program, it's always best..." - **PL2**	More check-ins needed with PLs	Transparency, trust, consistent communication, sense of community, continual guidance from CDCES, good communication, prioritizing participant privacy,	**Infrastructure:** PL as point of contact, recruitment, participant/PL testimony, community spaces
Incentives and Resources	What resources do [researchers] need to implement a PLDSMS program for Black men with type 2 diabetes? What financial incentives and disincentives do community collaborators and organizations have in implementing PLDSMS for Black men with type 2 diabetes?	"[For a health system] So I think in diabetes, if you could say look, this is going to improve your quality measures, A1Cs are going to be better, you know African American men are often hard to engage in programs... in pre diabetes is going to be hard, because prevention is not incentivized but because diet, A1C control is a quality measure and blood pressure is a quality measure that would be what you would need to target...It's certainly much cheaper than having nurse care management...unfortunately we've realized much more that you have to show that return on investment to get traction." - **Researcher 2** "At this point, you know from a financial perspective, I know there's work being done. Around having the work that community health workers do be billable, you know, generate revenue...I think there's a growing recognition in the medical community about the need to address the social determinants of health in their patients, and I think there's a recognition that ancillary providers, whether it's social workers or care managers or lay community health workers, are an integral part of addressing those needs, so I think that has provided some incentive." - **CC**	Diabetes self-management support (DSMS) not billable, lay health workers not integrated	Insurance coverage, improving quality measures, increased recognition of the social determinants of health	**Skills:** Staying informed, facilitation, cultural awareness, flexibility
Capacity for Organizational Change	What organizational changes are needed to implement a PLDSMS program in a health system?Who do you believe would support the necessary changes?	"So I think that there needs to be changes in the delivery system...rather than there being this referral system if diabetes education and support was part of the clinic or part of the primary care practice, I think it would cut down on a lot of the fragmented ways in which care is delivered and it would help the referral process. And that was sort of that wasn't me that was the work that I was involved in when I was at (university name). Our referrals to diabetes education were horrid. And once we tested putting a diabetes educator into the primary care practice on Diabetes Day, if you will. Things improved exponentially and then the health and then based on that the healthcare system took that on and agreed that this was how they were going to facilitate diabetes, education and support." - **Researcher 1** "So I mean there were a lot of stakeholders who needed to buy in, and it would be the same thing in any system. I think that the number one driver of the success in that model was that we had this physician champion and this particular physician, he was in charge of all the primary care physicians in the entire health system...he totally bought into the model and he was able to tell his docs know you're doing this, make it happen." - **Researcher 1** "So, I think places like the [health centers] and other federally qualified health centers, there are no barriers, those are the community based clinics that have been at the forefront of embedding community health workers into their practice. Where the resistance comes is more in the sort of corporate medical model, where it's just a different context and there's a continuing need to educate those providers in the corporate world about the need to have a more comprehensive approach." - **CC** "...it needs to be embraced or adapted into the for the service provision of the department and understanding what those requirements would be in order for it to be considered a value deliverable for the department...how would it pay for itself in terms of a good peer leaders or regularly and encouraged to be paid, so that they can have some value to their time and effort that they put in because it's a lot of emotional time." - **CDCES2**	Integration with healthcare system, paying peer leaders, billable DSMS, certification, preconceived obstacles; corporate model - resistance to lay health workers	Physician champion; federally qualified health center (FQHC) and community-based clinics - integrating lay health workers	**Infrastructure:** Alignment; supervisor - RN or social worker **Skills:** Interpersonal (supervisor)
Use of Technology	Is there a way that technology (such as internet or video visits) could be used to administer a PLDSMS program? Why or why not?	"...Zoom is not the best way. And I think it sort of goes back to the tailoring aspect you know if someone is uncomfortable with a video visit. Then they're expanding more energy on that, rather than what they should be focusing on. And you know we also don't know whether video videos are any more effective than just phones. Especially for people who struggle with technology...it's also very dependent on the population of people. You know, as more and more younger people get diabetes, more and more people will be more comfortable with using an app...using a website." - **Researcher 1** "...the huge advantage is that there's not the transportation barriers...we're doing all our peer monthly peer coach sessions by Zoom, and we're getting 100% participation, whereas we never got that before so I don't know...I think it might be harder to develop rapport...it's harder to to develop a sense of kind of group cohesion. On the other hand, you get better attendance and I do think, as we all learn how to get more comfortable with Zoom I think we're going to see increasingly that people are just relaxed." - **Researcher 2** "The whole concept of telehealth is a great one, for a lot of reasons, but also a very challenging model...I know that there are some among the patient population who either don't have access to technology or who are uncomfortable with the technology. You know it's funny, here I am at the [local health center], and we serve young people; we've seen a decrease in our no-show rate because we're doing telehealth...At the same time, older populations and myself included, I'm a Luddite and the technology is challenging. You're talking about working with an older population, so the use of that technology might be challenging, and, not to mention, the access piece. So, I guess, pros and cons but for the most part, I do think it creates an additional way to reach out to folks and an additional option for people who may have other barriers to accessing care." - **CC** "...I think it was we did have some issues with kids coming in the room. Yeah and spouses come in the room...And so it kind of depends on the privacy, and so I think it varies, you know if I can go in a room and my wife isn't there...So privacy from your family is an issue...I tried just getting some slides that we can pop up to say like when we're talking about your blood sugars and the graphs that are just all over the place. But when I draw, it helps me slow down and explain it better. So I hated I didn't like it, as I preferred as a as a speaker or as a teacher or facilitator I'd liked it better, but on the other hand, I couldn't work with this group in San Francisco San Diego's obviously so that was a plus. There is plus and minus but it helps to have people, somebody you're going to need somebody who can actually facilitate..." - **CDCES1**	Dependent on participant comfort, accessibility (Zoom), lack of community, limited privacy, managing virtual delivery	Dependent on participant comfort, accessibility (transportation), increases attendance, less judgmental, convenience	**Infrastructure:** Balance (in-person and online)
		I think, technology is a gift and a curse...a lot of communication is nonverbal, and you can see that and sense that. When you're in person, you get it. Virtually, you can get it as well just [there’s] still something that's missing that I don't think can just be replicated virtually like it is in-person, but it's a close second...what's easy to do is also easy not to do...So, you know, it's easy, it’s like 'hey it’s at our fingertips, boom, I'm on' or, 'you know what, I'm going to do something else right now,'...being able to be a part of something with guys that you may not have been able to participate with because of where we reside and being able to have a virtual means to pull everyone together is a positive as well. - **PL3**			

^a^Domains in the TICD Checklist (Flottorp, S. A., Oxman, A. D., Krause, J., Musila, N. R., Wensing, M., Godycki-Cwirko, M., Baker, R., & Eccles, M. P. (2013). A checklist for identifying determinants of practice: a systematic review and synthesis of frameworks and taxonomies of factors that prevent or enable improvements in healthcare professional practice. Implementation science : IS, 8, 35. 10.1186/1748-5908-8-35

^b^*PL* peer leader, *CC* community collaborator, *CDCES* certified diabetes care and education specialist

### Guideline factors

Guideline factors aimed to capture the feasibility and acceptability of implementing a PLDSMS program. As part of this feasibility and acceptability, participant perspectives on the importance of PLDSMS programs were also captured. One participant, who had nearly 15 years of experience working with PLs and who had experience as a diabetes researcher, felt that there were no established guidelines to help develop PLDSMS programs.

Another participant described themselves as a facilitator and believed that as a guideline, it is important to ensure that the environment is judgment-free. Others felt that peer-led diabetes education programs simply advise participants to make changes; they do not show them how to do it. The lack of continued support made them feel as though these programs were not needed. Education programs, in the absence of ongoing support, tend to generate no money and the health care system cannot support sustainability. Nevertheless, it was acknowledged that having a peer diabetes program was important, as the peers are people who have diabetes, look like the participants and are non-judgmental – they come from a place of understanding. Participants felt the program generally created space for relationship building, where thoughts were validated.

Some similarly shared that losing the rigidity often found in research may be valuable, but at the same time this level of flexibility can be detrimental in improving health outcomes. Hosting PLDSMS programs in community-based settings, compared to medical settings, offers a more relaxed environment for participant transparency.

*“Community. I feel like where the atmosphere is relaxed… In a medical center [it’s like] okay [is] this about medical*,* is this about this…but [if] you have an open*,* free environment then you can be free…it opens the door for them…”* (PL2).

### Individual collaborator factors

Building on program implementation feasibility and acceptability, individual collaborator factors sought to identify what skills peer leaders and diabetes educators needed to be successful. Participants shared that communication, patience, and excellent listening skills were key qualities every PL should have. PLs should also possess interpersonal skills and be willing to learn. To be a PL, you must be interested in learning, knowledgeable about the subject (i.e., T2D), and personable.

*“As a facilitator*,* your openness to learning. I think*,* you know and*,* again*,* it could be personality*,* it can be something else or whatnot*,* when you go into something you got to be open to learning new things*,* and not coming from a posture of you know it all…”* (PL3).

Others shared that to be a PL, one must have a level of discernment. Individuals must be willing to deviate from the structure; “blend the two.” Meeting the men where they are at really allows the PL to direct the conversation and leads to participant transparency.

*“The flow of it*,* the way that they wanted it to flow*,* don’t always work out like that…the format that we had*,* I thought that was a good format to follow*,* but that don’t mean that it’s going to always follow that way*,* especially once you get the men to really open up they’re going to kind of flow into what they think is important for them and what they need. So*,* you would sometimes go off the flow…”* (PL2).

### Patient factors

Understanding whether Black men with T2D would be receptive to participating in a PLDSMS program influences acceptability. Understanding where to recruit, similarly, influences acceptability. Participants stated that a selling point of the program is the ability for the men to share their results with one another. Some felt that if individuals want Black men to be more transparent, sometimes a more unstructured method of engagement is necessary. One participant shared that the program participants built a “strong community,” and they kept in contact with one another long after the program ended. Per the participants, the program works – Black men tend to be more willing to talk with other Black men who share similar experiences. Black men tend to keep things to themselves. A program that builds trust is vital; this level of trust has been broken in medical settings, primarily due to historical events. The PLDSMS program provides an opportunity to heal.

*“…having a group like this to be able to talk about those things…have others to validate those feelings…using that as a conduit to heal and also to move past it…as we talked through these things. So I think it’s extremely important to have programs like this in place*,* because without them I just feel that we’re not engaging other circles that we may be in to talk about these issues that have such a huge impact on our lives. Breaking down the social norms of African American/Black men and allows for discussions about health outside of the medical setting since there is distrust in the medical community. Actively participating in conversations about their care*,* healing process that happens mentally and physically from being misunderstood by community members and health care teams over the generations.”* (PL3).

In terms of how feasible it is to administer a PLDSMS program for Black men with T2D, the overall sense was that this program allowed the men to really be themselves, where they felt comfortable sharing their truth. It was like a brotherhood.

*“…we became more unified*,* because now we feel like we’re like a brotherhood because we all dealing with the same issues*,* so we all understood we all in this thing together. It became like a brotherhood thing*,* we exchanged numbers…”* (PL2).

The program addresses masculine norms among Black men while offering a safe space for participants to have discussions with other older Black men living with T2D.

*“Strangely enough*,* [the program] certainly avoids the people shutting up because they don’t want to talk about things with people of another race…I think that was a very good thing about doing it with Black men as opposed to all men in general…it’s like you’re supposed to be the strong guys there*,* and you got something here that can tear that down and some of the things that can help that [or] can at least slow down the diabetes itself*,* which is really the cause of the problems.”* (PL1).

Based on experience, one participant felt that many of the men dissociated from their condition, particularly since they felt young and healthy, making the program even more essential. Hearing from other men who were going through somewhat similar experiences can shift mindsets. Additionally, the importance of creating a safe space so that men can be vulnerable and not ignored is critical. CDCES2 stated:

So can I go back to the first part…of your question, because it reminded me of the shame stigma point that men who may be at the door of complication or loss or some kind of illness- stroke heart attack, whatever it is- the not wanting to look weak… one of the gentleman talked about voicing his shame for having problems with diabetes and not being worthy feeling like he was out of control and had no ability to make any changes and he took the risk of being vulnerable to speak it and was able to then find a safe ground because of the platform of everyone is just going to be open and and no one was allowed to just like jump on anyone or talk to tell you that you can’t do that way you can’t feel that way, etc., so the ground rules literally provided the safe place for him to be vulnerable….you could hear it when he came back to subsequent groups that he felt more empowered to speak.

In recruiting Black men to participate in PLDSMS programs, churches seem to be a good starting point. One participant stated that working with doctors to share information may also be an effective recruitment strategy. Recruiting in places where Black men frequent, such as churches and barbershops, is valued, and gives participants the freedom to share their story.

*“Yes*,* there are more women in the churches than men*,* but particularly those men who have diabetes and who are suffering from it*,* they could very well be at the [churches? ]. Well*,* they would make good candidates. If the information that it’s available could be spread through doctors*,* then I think that would help also.”* (PL1).

Others agreed that churches look to be ideal to host PLDSMS programs, but unfortunately not as many men attend church so this may not be the best setting. They suggested that barbershops may be more suitable, and hosting information sessions where PLs share their experiences may also positively impact recruitment. Study participants further suggested that PLs can promote the program as a small support group, allowing men to maintain their privacy and masculinity. PLs can remove social stigma and negative expectations to create a safe space that challenges social norms and societal expectations of what is available for Black men. The program may encourage people living with T2D to speak with family and friends who are also living with T2D.

### Professional interactions

The methods and frequency of communication between participants and staff play a role in program sustainability. One study participant expressed that communication of research timelines, expectations, and resources was critical to running a peer-led program. Consistent and varied communication and initial training with PLs are crucial. In addition, listening to what the community is asking for and incorporating that into the study, as well as building trust with community members, are important. DSMS based on empowerment is tailored to the patient, for example, but there is still work to do. There is a need to better understand what the community needs, and not rely on what researchers think is important.

*“…I would say what has worked well… making sure that people have a voice. Everybody*,* …not just the people doing the study*,* but the people in the community. I think that we need to be better at understanding what the community needs. What they perceive as important. In comparison to what we think is important. Being transparent around timelines around incentives. If it is a research study*,* being very*,* very clear about the research processes that are inevitable in a study.”* (Researcher 1).

Study participants observed good communication between PLs and the program staff, but suggested more post-session discussions on PL skills. They believed that increased check-ins with program participants outside of sessions would have been beneficial. Participants felt that the program organizers and facilitators cared about their diabetes self-management and wanted them to do better. Debriefing sessions would have, however, facilitated continuous communication of shared goals.

*“I think the communications process with the team was handled pretty effectively…I think [program manager] did a great job of communicating things with us. You know*,* [program manager] has a special place in my heart. She’s great. Love [program manager]*,* and I think she prepared us*,* you know very well*,* to be able to run those classes*,* facilitate those classes. I think*,* maybe*,* the only thing I would say is that one thing that could have been done is me and [fellow peer leader] could have communicated more just between us…Just the insights that we’re getting from it…maybe…. a 15 minute debrief after the meetings or prior to*,* just so that you know we’re staying sharp on what we’re doing and making sure that we’re focused on the goal going into it. That would be the only thing I would kind of recommend*,* but overall*,* I think the communication was very effective between the staff and the peer leaders.”* (PL3).

Adjusting the structure of PLDSMS programs, particularly the method of communication, may be necessary to meet participant needs. Although reference was made to holding 15-minute debriefs with the research team to ensure peer leaders were on task, respecting the peer leaders’ time was recognized.

*“I know we did have a couple of times where we would assess a meeting after we had it*,* but it wasn’t always*,* and I think some of it had to deal with the fact that we’re on it for an hour and a half*,* so… and [program manager] was respecting our time*,* knowing that*,* you know*,* we have lives that we’re living as well…”* (PL3).

### Incentives and resources

The PLDSMS program personally helped PLs with their diabetes self-management. PLs considered this to be enough of an incentive. The opportunity to give back as a PL made participation most meaningful.

*“The rewards that you receive and just those things alone that money can’t buy…it’s more about the reward that you giving and receiving than the money*,* because you’re helping people save lives… if we don’t take care of diabetes or don’t have education*,* we’ll die. That’s the way many of us have died*,* between dying*,* amputations*,* and blindness because of lack of information…I’ve learned a lot at that. I know I was a peer leader*,* but I also was a participant. I felt like I sat on both sides*,* because one of the greatest things I did learn is that I have been taking my medication wrong. Now*,* what if I didn’t participate in this*,* I would still be taking my medication wrong for some more years.”* (PL2).

Training and recruiting are paramount to running a PLDSMS program. One participant, from previous experience with a prediabetes peer coach program, found that it was hard to retain PLs over time due to their own time constraints, even when participation was incentivized. These sentiments diverge from what the PLs shared in this study. Regarding resources, participants expressed that people (i.e., champions) are essential if you are aiming to set up an ongoing DSMS program. Space and money, if needed, and commitment are also important.

### Capacity for organizational change

The researchers, CC, and CDCESs were asked about the organizational changes needed to implement a PLDSMS program in a health system or community setting. A significant reported challenge was effective integration with the healthcare system. Participants explained that due to the structure of the healthcare system, leadership is often resistant to lay health workers and to change. Larger/private institutions have more barriers relative to Federally Qualified Health Centers (FQHCs), for example. Moreover, PLDSMS is not billable and PLs typically do not get paid for their time. There needs to be better integration between community-based diabetes education programs and the medical management of individuals’ diabetes.

Support from someone in leadership, with influence and power, who believes in the program and is positioned to make changes in the medical system, is necessary. In addition to being adequate in size, systems may benefit from having physicians who buy-in for program adoption to be effective. Physicians tend to support PLDSMS programs since they improve health outcomes without adding to physician workload; the role of the PLs is to help with action planning. Participants further expressed that PLs need a supervisor, and that barrier is difficult to overcome. Social workers or nurses may be able to supervise PLs, but those in these roles are already overextended. Input from CDCESs would be helpful to determine how to make a program sustainable, but there are no clear solutions yet. Having an infrastructure to adopt and run the program, financial support and other resources, and hiring PLs so the program can continue, are warranted.

One participant’s previous experience adding a CDCES into primary care improved the referral process, and the process was later officially adopted at their institution. Another participant had experience with prior church-based programs. They communicated that the programs would have been more sustainable if the churches took ownership of the process, but the churches preferred to have the researchers do everything. According to this participant, for DSMS programs to be effective in churches, church leadership must make the program a priority; it was the priority of the research team, not the church.

### Use of technology

While technology can be beneficial, there are potential drawbacks. Some felt that Zoom was not the best way to deliver PLDSMS. They noted that some program participants are uncomfortable with video visits, yet many programs have transitioned to a virtual format. Videos may not be more effective than phones, particularly for those who struggle with technology. Additionally, individuals oftentimes multi-task while on Zoom.

Despite the potential to engage in other activities, some participants viewed the platform favorably, as it provided an opportunity to participate, something that would not have been possible if the program had been offered in person. They appreciated the ease and convenience of Zoom, particularly as it facilitates participation for those who have busy work schedules or transportation challenges. Although in-person programs provide a unique experience that cannot be replicated using Zoom, Zoom, nonetheless, is convenient, as individuals can join from any location, allowing for a larger number of participants. Interacting with others in a physical setting, however, where participants’ energy is more tangible, is equally important. Study participants concluded that a hybrid program would be well-received.

## Discussion

This qualitative study aimed to identify whether the practices of an evidence-based intervention was designed with the internal organizational policies and priorities / frontline providers in mind. Our objective was to assess commitment and organizational capacity, and identify top-down and effective implementation strategies that empower providers. Tailoring interventions that acknowledge cultural sensitivity and align with the values, perspectives, and preferences of the target population is important for success. In our study, collaborating with community social institutions, such as barbershops, was identified as a facilitator. Barbershops have long been recognized as trusted recruitment spaces for communities of color that are at elevated risk for chronic conditions [23]. In addition to facilitating recruitment, barbershops provide a safe space for Black men to openly discuss health-related topics without the judgment or discomfort that tends to be associated with medical settings.

Our study conclusions align with previous findings that emphasized the role of barbershops in fostering mental well-being among Black men [24]. Developing PLDSMS programs that partner with community leaders in these spaces promotes safety, engagement, and participation. In our study, churches were similarly identified as safe spaces that could be leveraged for health promotion interventions, particularly when there is buy-in from leadership. In one study aimed at refining the recruitment process for a resilience-based diabetes self-management education program for Black adults, there was interest in attending a church-based program if there was buy-in from the pastor to help build trust and enhance recruitment and retention efforts [25]. A noted barrier to program sustainability was the lack of adequate support from community collaborators. To address any lack of commitment from church leadership, it is critical that members of the research team engage community leaders during the early stages of the development process. Demonstrating how the program impacts the health of the congregation, and the broader community, will help support sustainability.

Flexibility is another key determinant of PLDSMS program success. Programs should be guided by evidence-based frameworks, but must also be amenable to participants’ preference of the setting, time, and form of communication. In our study, peer support was emphasized as a major facilitator. PLDSMS programs should give PLs the autonomy to tailor their support based on the needs of participants, particularly as needs may vary over the course of the program. PLs, who are often leaders in their own community, can function as advocates for future program development and recruitment.

As a result of prior injustices, Black men have historically been wary of the medical community. This apprehension was acknowledged as an initial barrier to participation. Although PLs in our study cited the mental and physical health benefits they received from having participated in a PLDSMS program, and felt that financially incentivizing their participation was unnecessary, peer-led programs should strongly consider offering financial incentives for PLs. Recruiting and retaining older Black men to participate in peer-led support programs requires thoughtfulness. Participation from PLs, who have a similar demographic background as the participants and who share the same medical condition, enhance discussions around diabetes management. Participants in our study reported feeling comfortable sharing their experiences. In subsequent designs of the program, PLs should receive skills training in relationship-building and emotional intelligence to foster a safe space for open dialogue. Such training would further empower PLs.

Lastly, peer-delivered interventions offered in person or virtually should lean on community preferences, comfort, and accessibility. Identifying participant needs via a thorough needs assessment prior to program development and implementation is critical to ensure participant needs are being met. Assessing participant beliefs and knowledge using theoretical models like the Health Belief Model [26] can further program design and importantly lead to early and continued adoption.

### Limitations and strengths

There are some notable limitations, but also strengths. Participants did not receive the interview guide or transcripts of the interviews, but data saturation was discussed during data collection, where the participants were largely homogenous, and the research question more narrowly focused. Additionally, the interview guide was not pilot tested, however, the questions were moderately modeled after a pre-existing interview guide [27]. Another notable limitation is that PL1 experienced health complications that inhibited his ability to effectively answer questions, significantly shortening the length of the interview and quality of his responses. The study did benefit from having two additional PLs participate to bolster findings. Of note, PL perspectives were largely reflective of having participated in a PLDSMS program led by the research team.

Another limitation was that the study was limited to the perspectives of those who lived in Metro Detroit, an urban area that cites T2D as the 8th leading cause of death, mirroring the national ranking [28, 29]. Given this geographic specification, perspectives may be more reflective of those residing in similar environments. Nonetheless, some of the participants had a breadth of experience through collaboration with individuals working and living in various parts of the US, negating what may be perceived as reduced generalizability.

In this study, the interviewer had pre-existing personal and/or professional relationships with the interviewees, which may have biased the way in which she asked the interview questions and consequently how the interviewees responded. Despite this, building trust with the participants cannot be overlooked and is vital in nurturing a safe environment for participant transparency.

## Conclusion

Guidelines and structure are important characteristics for the successful development and implementation of peer-led support programs, but flexibility within the structure is paramount, as peer leaders must adapt to the needs of participants to create an environment that fosters increased dialogue. For older Black men, who have lower rates of participation in research due to medical mistrust triggered by historical events, this level of flexibility is crucial in building trust. To effectively recruit older Black men as participants in PLDSMS programs, efforts must be targeted.

Partnering with community members during the development and implementation phases, and providing ongoing feedback to the PLs and staff after each session, may aid in program sustainability. There is an opportunity to scale the program by integrating it into larger healthcare institutions. Structural barriers that include issues around billability, must be addressed. Physician champions, inclusive of healthcare executives, can help to advocate for inclusion of PLDSMS programs in healthcare systems.

Although providing financial incentives for PLs carries some value, the knowledge and awareness gained from participation are far more valuable. It is essential to recognize the importance of factors outside monetary compensation that motivate participation. While Zoom affords participants added convenience, an in-person setting offers an experience that cannot be replicated virtually. In future iterations of the program, a hybrid model may be warranted, where complete engagement and relationship building remain central. Future research should examine the feasibility and acceptability of a hybrid model, as well as its impact on health outcomes, relative to models that are completely virtual or completely in person.

Findings will be used to inform future iterations of PLDSMS for Black men with T2D and will assist other practitioners in the development and implementation of diabetes peer-led programs tailored for Black men.

## Supplementary Information

Supplementary Material 1.

## Data Availability

Data are provided within the manuscript.

## Abbreviations

CC: Community collaborator
CDCESs: Certified diabetes care and education specialists
PI: Principal Investigator
PLs: Peer leaders
PLDSMS: Peer-led diabetes self-management and support
RADaR: Rigorous and accelerated data reduction
T2D: Type 2 diabetes
TICD: The Tailored Implementation in Chronic Diseases
US: United States
