# Supplementary material for: The Michigan men’s diabetes project: perspectives on a peer-led diabetes self-management and support intervention for Black men with type 2 diabetes
Source: BMC Health Serv Res. 2024 Dec 18;24:1612. doi: 10.1186/s12913-024-11884-2 (PMC11654413; doi:10.1186/s12913-024-11884-2)
Supplement: Supplementary file 1 — Supplementary Material 1. [file 12913_2024_11884_MOESM1_ESM.docx]

**Peer Leader Zoom Interview Guide**

**PERCEPTIONS OF FACTORS THAT IMPACT THE IMPLEMENTATION OF A PEER-LED DIABETES SELF-MANAGEMENT EDUCATION AND SUPPORT INTO A HEALTH SYSTEM**

This document is meant as a guide only. It is our intention that the participant will direct the flow of conversation. This document is not meant as a script, but as a tool for the moderator to make sure that all areas of discussion are covered. Questions formatted in *italics* are suggested probes that may or may not be necessary.

**Welcome and Introduction**

The moderator introduces themself.

*I want to thank you for taking the time out of your busy day to participate in this Zoom interview. We have invited you to this interview to get your opinions about factors that impact the implementation of a peer-led diabetes self-management support specifically for Black men with type 2 diabetes. In a peer-led diabetes self-management support program, people with diabetes are trained as peer leaders to facilitate support sessions for other people living with diabetes. For this specific intervention, when we talk about peer-led diabetes self-management support, we will be talking specifically about Black men with type 2 diabetes being peer leaders for other Black men with type 2 diabetes. These support sessions can be held virtually or in person, last about 1-1.5 hours in time, and occur on a weekly, monthly, or quarterly basis depending on the needs of the group members. Throughout this interview, I will refer to peer-led diabetes self-management support as PLDSMS.*

*I will be asking questions about some of the important issues you raise. We plan to use your feedback to improve future diabetes programs for Black men with diabetes.*

**Order of Business**

Introduce how the discussion will proceed.

*This Zoom interview will last approximately 1 to 1 ½ hours. After the Zoom interview, you will receive a gift of $30 in the form of a check or gift card to be mailed to your home address in appreciation for your participation. You may end the interview at any point.*

**Explain Zoom Interview**

Explain the concept behind ‘Zoom interview’

*A Zoom interview is another way of asking questions to gather information-I will ask some questions, but would like/hope that you will do most of the talking. This is an opportunity for you to share your thoughts, feelings, and opinions about how peer-led diabetes self-management education can be structured for implementation in the health system.*

*There are no right or wrong answers. We are interested in hearing about your point of view.*

**Zoom Interview Guidelines**

*There are a few guidelines for the Zoom interview.*

- *We value your openness – both positive and negative opinions are valuable to us. Sometimes the negative opinions can be even more helpful than the positive because they help us know what to change. I want to hear your opinions, so don’t be afraid to share.*
- *We’re interested in your opinion—At the same time, if there are any questions you do not wish to answer, feel free to just ‘pass’.*
- *Our main job, though, is to listen, and to make sure that you have a chance to be heard. Every effort to keep the discussion on track. If too much time is being spent on one question, or on something off-topic, we may move the conversation along so we can cover all the questions.*

**Confidentiality issues**

*We will be on a first name basis today, but there will not be any names attached to the comments in the final reports. You may be assured complete confidentiality.*

*This Zoom interview will be recorded to be sure we don’t miss anything. We will obtain verbal permission from you prior to recording the interview. Later, we will transcribe the content of the tape. Your identity will be kept completely confidential. The audio-tapes will be destroyed once we type the Zoom interview, and any names will be removed from the transcripts.*

**Interview questions**

*Okay, now I am going to start the Zoom interview with a general question:*

1. **Rapport Building**
   1. **Could you begin by telling me a little about your role and how long you have worked as a peer leader?**
2. **Guideline Factors**
   1. **Recommendation**
      1. **Clarity**
         1. **What are the guidelines for implementing a PLDSMS program?**
            1. Are the guidelines clear and understandable?
            2. *Is there enough detail provided to enable peer leaders to successfully implement the program?*
      2. **Quality of evidence and strength of recommendation**
         1. **In your opinion, are PLDSMS programs needed? Why or why not?**
   2. **Cultural Appropriateness**
      1. **Is PLDSMS appropriate for the culture of medical settings and community settings? Why or Why not?**
         1. *Does it align with values, beliefs, behaviors, and goals of these settings?*
   3. **Recommended clinical intervention**
      1. **Feasibility**
         1. **In general, how feasible is it to administer a PLDSMS program for Black men with type 2 diabetes?**
            1. *What would make it difficult? What would make it easy?*
            2. *In a community setting? In a healthcare setting?*
3. **Individual Collaborator Factors**
   1. **Knowledge and Skills**
      1. **Awareness and familiarity with the recommendation**
         1. **What skills do peer leaders need to have to implement a PLDSMS program for Black men with type 2 diabetes?**
            1. *Do you think you have those skills?*
            2. *Do you interact with other peer leaders in a way that would facilitate or hinder implementing a PLDSMS program for Black men with type 2 diabetes?*
   2. **Cognitions**
      1. **Expected outcomes**
         1. **How do you think a PLDSMS program affects health outcomes for Black men with type 2 diabetes?**
4. **Patient Factors**
   1. **Patient needs**
      1. **How are the needs of Black men prioritized in a PLDSMS program compared to other diabetes self-management support (DSMS) programs?**
   2. **Patient preferences**
      1. **Do you think Black men with type 2 diabetes want to receive PLDSMS? Why or why not?**
   3. **Patient behavior**
      1. **Why do you think Black men may be interested in participating in PLDSMS programs?**
      2. **What might prevent Black men from participating in PLDSMS programs?**
         1. *Describe the barriers that may hinder black men from participating in PLDSMS?*
   4. **Is PLDSMS culturally appropriate for Black men with type 2 diabetes? Why or why not?**
      1. *What can peer leaders do to make PLDSMS more culturally appropriate for Black men with type 2 diabetes?*
   5. **Is PLDSMS accessible for Black men with type 2 diabetes?**
      1. *What can peer leaders do to make PLDSMS more accessible for Black men with type 2 diabetes?*
5. **Professional Interactions**
   1. **Communication and influence**
      1. **What is the communication process among team members while running a PLDSMS program for Black men with type 2 diabetes?**
         1. *What works particularly well? What may improve communication?*
         2. *How frequently do team members communicate? What do team members primarily use to communicate (phone, email, text)?*
         3. *What topics do team members communicate the most about?*
      2. **What is the communication process with participants?**
         1. *What works particularly well? What may improve communication?*
         2. *How frequently do team members communicate with participants? How do you communicate with participants (phone, email, text)?*
         3. *What topics do you communicate with participants about?*
   2. **Team Process**
      1. **What outside groups do peer leaders need to communicate with to be able to implement PLDSMS for Black men with type 2 diabetes?**
         1. What hinders successful communication with these outside groups? What supports successful communication with these outside groups?
   3. **Referral Process**
      1. **How can peer leaders support Black men with type 2 diabetes to learn about and enroll in PLDSMS?**
         1. What barriers, if any, may you have in this process? What may help remove these barriers?
         2. If there is no referral process, how do you think peer leaders can advocate for initiating this process?
6. **Incentives and Resources**
   1. **Availability of necessary resources**
      - 1. **What resources do peer leaders need to implement a PLDSMS program for Black men with type 2 diabetes?**
           1. *Resources may be financial, equipment and supplies, and technical capacity. Are these resources available?*
   2. **Financial incentives and disincentives**
      1. **What financial incentives and disincentives do peer leaders have in implementing PLDSMS for Black men with type 2 diabetes?**
         1. *Financial incentives and disincentives are related to money.*
   3. **Nonfinancial incentives and disincentives**
      1. **What non financial incentives and disincentives do peer leaders have in implementing PLDSMS for Black men with type 2 diabetes?**
         1. *Non financial incentives and disincentives are not related to money.*
   4. **Assistance for clinicians**
      1. **What type of assistance do you believe peer leaders need to implement PLDSMS?**
         1. *Assistance may be related to patient information, decision support or clinical supervision.*
         2. *Do peer leaders have access to this type of assistance? Why or why not?*
7. **Capacity for Organizational Change** *(this section only applied to the researchers, community collaborator, and certified diabetes care and education specialists)*
   1. **Mandate, authority, accountability**
      1. **What organizational changes are needed to implement a PLDSMS program in a health system?**
         1. *Who has the mandate and authority to make these changes?*
         2. *Who is accountable, to whom, and how?*
         3. *How can diabetes educators support these changes?*
   2. **Regulations, rules, policies**
      1. **How do internal or external organization regulations, rules or policies facilitate or hinder implementing a PLDSMS program in a health system?**
   3. **Assistance for organizational change**
      1. **Who do you believe would support the necessary changes?**
         1. Why do you think these people would support the necessary changes?
      2. **Who opposes the necessary changes?**
         1. Why do you think these people would oppose the necessary changes?
8. **Use of technology**
   1. **Some diabetes education and support programs are offered virtually. Is there a way that technology (such as internet or video visits) could be used to administer a PLDSMS program? Why or why not?**
      1. How might technology be a benefit for the peer leader? How might it be a disadvantage?
      2. How might technology be a benefit for the participant? How might it be a disadvantage?
   2. **Internet and technology knowledge**
      1. Do you think peer leaders have the internet and technology knowledge to facilitate a PLDSMS program virtually?
      2. Do you think participants of the program have the internet and technology knowledge to participate in a virtual PLDSMS program?
   3. **Internet and technology access**
      1. Do you think peer leaders have access to the technology and internet needed to facilitate a PLDSMS program virtually?
      2. Do you think participants have access to the technology and internet needed to participate in a virtual PLDSMS program?

***Any additional comments?***

*Thank you for sharing your experiences with us and for the honest feedback regarding your opinions about peer-led diabetes self-management support for Black men with type 2 diabetes. This information will be very useful for designing future programs. Are there any other comments or thoughts that you have which were not voiced during the interview?*
